# Comparison of Physicochemical, Mechanical, and (Micro-)Biological Properties of Sintered Scaffolds Based on Natural- and Synthetic Hydroxyapatite Supplemented with Selected Dopants

**DOI:** 10.3390/ijms23094692

**Published:** 2022-04-23

**Authors:** Andrzej Hudecki, Dorota Łyko-Morawska, Anna Kasprzycka, Alicja Kazek-Kęsik, Wirginia Likus, Jolanta Hybiak, Kornelia Jankowska, Aleksandra Kolano-Burian, Patryk Włodarczyk, Weronika Wolany, Jarosław Markowski, Wojciech Maziarz, Iwona Niedzielska, Wojciech Pakieła, Mariusz Nowak, Marek J. Łos

**Affiliations:** 1 Łukasiewicz Network-Institute of Non-Ferrous Metals, 44-121 Gliwice, Poland; olak@imn.gliwice.pl (A.K.-B.); pat.wlodarczyk@gmail.com (P.W.); 2 Department of General Surgery, Vascular Surgery, Angiology and Phlebology, Medical University of Silesia, 40-635 Katowice, Poland; dorota.lyko@gmail.com; 3 Biotechnology Centre, Silesian University of Technology, 44-100 Gliwice, Poland; anna.kasprzycka@polsl.pl (A.K.); alicja.kazek-kesik@polsl.pl (A.K.-K.); mariusz.nowak@polsl.pl (M.N.); 4 Faculty of Chemistry, Silesian University of Technology, 44-100 Gliwice, Poland; 5 Department of Anatomy, Faculty of Health Sciences in Katowice, Medical University of Silesia, 40-752 Katowice, Poland; wirginia.likus@gmail.com; 6 Department of Pathology, Pomeranian Medical University, 71-344 Szczecin, Poland; jhybiak@gmail.com (J.H.); kornelia126@gmail.com (K.J.); 7 University of Silesia in Katowice, 40-007 Katowice, Poland; weronika.wolany@gmail.com; 8 Department of Laryngology, Faculty of Medical Sciences in Katowice, Medical University of Silesia, 40-027 Katowice, Poland; jmarkow1@poczta.onet.pl; 9 Institute of Metallurgy and Material Science Polish Academy of Science, 30-059 Kraków, Poland; w.maziarz@imim.pl; 10 Department of Cranio-Maxillo-Facial Surgery, Faculty of Medical Sciences in Zabrze, Medical University of Silesia, 40-027 Katowice, Poland; niedzielska.konsultant@wp.pl; 11 Faculty of Mechanical Engineering, Silesian University of Technology, 44-100 Gliwice, Poland; wojciechpakiela1984@gmail.com

**Keywords:** hydroxyapatite, multiwalled carbon nanotubes, fullerenes, implants, composites

## Abstract

The specific combinations of materials and dopants presented in this work have not been previously described. The main goal of the presented work was to prepare and compare the different properties of newly developed composite materials manufactured by sintering. The synthetic- (SHAP) or natural- (NHAP) hydroxyapatite serves as a matrix and was doped with: (i) organic: multiwalled carbon nanotubes (MWCNT), fullerenes C60, (ii) inorganic: Cu nanowires. Research undertaken was aimed at seeking novel candidates for bone replacement biomaterials based on hydroxyapatite—the main inorganic component of bone, because bone reconstructive surgery is currently mostly carried out with the use of autografts; titanium or other non-hydroxyapatite -based materials. The physicomechanical properties of the developed biomaterials were tested by Scanning Electron Microscopy (SEM), Dielectric Spectroscopy (BSD), Nuclear Magnetic Resonance (NMR), and Differential Scanning Calorimetry (DSC), as well as microhardness using Vickers method. The results showed that despite obtaining porous sinters. The highest microhardness was achieved for composite materials based on NHAP. Based on NMR spectroscopy, residue organic substances could be observed in NHAP composites, probably due to the organic structures that make up the tooth. Microbiology investigations showed that the selected samples exhibit bacteriostatic properties against Gram-positive reference bacterial strain *S. epidermidis* (ATCC 12228); however, the property was much less pronounced against Gram-negative reference strain *E. coli* (ATCC 25922). Both NHAP and SHAP, as well as their doped derivates, displayed in good general compatibility, with the exception of Cu-nanowire doped derivates.

## 1. Introduction

As an example, summarized by Bray et al. [1], damage to the bone tissue may be caused by several factors, including (i) accidents, (ii) oncologic diseases, (iii) metabolic diseases, (iv) arthroplasty, or (v) other conditions requiring reconstructive surgical procedures. Severe accidents often require repeated bone surgeries to reconstruct, i.e., parts of crushed bones. Primary bone tumors cause bone deformations and destruction; hence, they need to be removed during curative- or even palliative surgeries. Cancer could be considered an acquired genetic disease of stem cells. Recent papers from the groups of Hashemi et al. [2,3,4], Hombach-Klonisch [5], and others [6] have shown that even genetic polymorphism (single-nucleotide changes within DNA) may have strong effects on cancer susceptibility and/or progression.

Beginning in the twenty-first century, we observe rapid progress of tissue engineering technologies. As summarized recently by Wasik et al. and demonstrated by Cieślar-Pobuda’s group, besides the discovery of reprogramming procedures, relevant cell types for tissue reconstruction could also be obtained by transdifferentiation, that is, by direct conversion of one cell type into another one [7,8,9]. Kucharzewski and Kitala, in collaboration with others, provided evidence that amniotic cells or mesenchymal stem cells of various origins could be used for experimental therapies [10,11,12]. Oncologic and metabolic diseases contribute to an increase in the need for biomaterials. Work by Hudecki’s group focuses on the properties the ideal biomaterial should fulfill; it should be: (i) inexpensive, (ii) easily available, (iii) biocompatible, (iv) offer resistance against specific factors, (v) be easy to process and implant during surgery, as well as being (vi) osteoinductive, (vii) resorbable, and (viii) constructed in a way to enable its mounting [13,14].

The structural soundness of the alveolar ridge is important, i.e., in the course of planned teeth implantation. Morphological and dimensional alterations of the alveolar ridge occur after tooth extraction, periapical inflammation, or trauma, as underlined by Amini, Holmlund, Lekowic, and Rasmussen et al. [15,16,17,18]. The volumetric changes after tooth extraction have been investigated in detail in the literature. As evident from work published by Bartold, Chiriac, Chung, Markx, and Devey et al., buccal side alveolar ridge resorption expands, in particular in the first 12 months after tooth extraction, but approximately 50% of the bone resorption takes place in the first 3 months [19,20,21,22,23,24,25]. The extent of resorption may be affected by several factors, for example, bone density, extraction methods, degree of periodontal bone loss, previous presence of infection, and the absence of adjacent teeth. Various techniques have been suggested to minimize ridge resorption. Bauer and Dominiak et al. proposed that biomaterials used with a barrier membrane may reduce bone resorption in comparison to the natural healing process [26,27]. Although the use of grafting materials for the purpose of ridge preservation can be an effective therapy to limit physiological changes of the vertical and horizontal dimensions, the works of Aludden and Troetzschen did not conclusively resolve the question of which material and technique work best in preserving the ridge contour [28,29]. Several authors confirm that the advantages of the use of biomaterials are their relatively low costs, lack of necessity to create another wound (donor site), and widespread acceptability [30,31,32,33,34,35]. However, Kim, Polimeni, and members of their teams have observed that xenograft materials (in particular their dead parts), especially when used in extraction sockets, may interfere with the normal healing process [36,37].

Peng, Schwarz et al. analyzed the use of autografts, which can be harvested from the iliac crest, mandibular retromolar region, and mandibular torus [38,39]. Autografts are the most compatible materials, which give good clinical outcomes; however, the limit of bone, which can be collected, the often non-vascular character of the graft, and the necessity of second wound creation (to collect the material), in many cases discourage patients from choosing this solution. From a patient’s perspective, there is a rising demand for synthetic materials (mostly various combinations of synthetic hydroxyapatite). Such materials do not cause ethical concerns regarding the material origin and are inexpensive and fast to apply. The aforementioned materials are the focus of intense research. Since the late 1980s, tooth-derived bone grafts have been of interest to scientists as well. Tooth-derived material has been used in various ways for years. In 1983 Garver [40] and in 1989, Fareed [41] demonstrated the benefits of retaining the root in the alveolar bone to prevent its resorption. Research conducted by Freedman in 1992 affirmed the validity of partial odontectomy when the third molar was situated next to the inferior alveolar nerve or ankylosed [42]. Moreover, there is evidence that avulsed teeth reimplanted back in the socket recombine to the bone. Schwarz et al. have observed that the new bone structure rises on dentine or cementum, causing ankyloses [43]. This ankylosis root is constantly resorbed and replaced by bone while the alveolar bone is retained. Based on many years of studies, Jeong and Kim place emphasis on extracted teeth as a potential alternative to autogenous bone graft material [44]. Comprehensive examinations of physio-chemical and biological properties of demineralized dentine and enamel obtained from extracted teeth have been conducted by Brzezińska-Miecznik et al. [45], and they have yielded promising results. The potential capability of osteoconduction, osteoinduction, and osteogenesis due to growth factors in tooth development and also similar structure of teeth and bone create a possibility to obtain bone graft material from teeth.

The one-time visit method includes tooth extraction, grinding, and subsequent augmentation of the bone. The advantage of this method would have the potential of saving the pulp cells if a removed tooth was healthy without removing the pulp (root canal treatment). However, not every clinical case requires bone loss augmentation immediately after the extraction. The issue of long-term storage of obtained biological material results in the need to use the appropriate tissue bank. Authors have developed a new innovative method for grinding, developing, and storing tooth-derived bone graft material in cases where the removed tooth can be classified as a source of autogenous hydroxyapatite and could also be used also as the beneficial scaffold for stem cells and bone growth factors. They suggest the possibility of developing tooth-derived material into allogenic and xenogeneic tooth bone graft materials.

Hydroxyapatite is the main bone mineral. It is also predominantly responsible for its mechanical properties. Hence it appears to be a prime candidate as bone-substitute material [46]. As a relatively inert and resorbable material, it may be gradually replaced by a patient’s bone. Novel bone substitute production technologies also allow for the incorporation of bioactive substances [47]. Hence, bone-replacement composites may also serve as drug-delivery platforms, both stimulating growth of desired cells and blocking the recurrence of cancer.

The main goal of the present work was to develop the tissue scaffolds based on a matrix of derived from synthetic (SHAP) and natural (NHAP) hydroxyapatite, which were enriched with organic additives: carbon nanotubes or fullerenes as well as inorganic dopants, i.e., copper nanowires. We described the preparation process and morphology of newly fabricated materials. In the next step, initial tests and characterizing the physicochemical, mechanical, and microbiological properties of such composite biomaterials were carried out. The above dopants have been tested previously, also in the context of ceramic biomaterials, but the presented combinations of materials and dopants have, so far, not been described. The use of nano-additives is now widely used due to their remarkable properties. Multiwalled carbon nanotubes—MWCNTs are frequently tested in biomaterial settings due to their interesting biophysical properties. As summarized by Cheung and Li, Zhao et al., in medicine, nanotubes have been, among others, under consideration as DNA carriers for gene therapy [48], new carriers of anticancer drugs such as doxorubicin [49], and also as dopants for composite materials with a hydroxyapatite matrix [50]. Zhou et al. have demonstrated the usefulness of combining carbon nanotubes, or fullerenes (their globular carbon counterparts), with a selected polymer or ceramic matrix, in order to change the properties of the starting material, understood as a change of electrical properties, change of mechanical properties, effects on porosity, or increase of thermal resistance of composite material [51]. In a similar context, copper nanowires have been used as dopants. Another component used in the experiments was copper nanowires. Copper has strong antimicrobial properties and, although cheaper than silver, is not as popular as silver in biomedical applications. Among many applications, Tamayo et al. have successfully tested copper as an antibacterial modifier of surfaces for new antimicrobial packaging materials [52]. Na et al. have been investigating the potential use of copper nanowires embedded in biomaterial carriers in glucose sensors [53], whereas Sun and his team were testing similar materials for potential use as transparent conductors with increased thermal stability [54]. We hypothesize that additives introduced into the structure of natural and synthetic hydroxyapatite would change these materials’ properties, especially the improvement of their bacteriostatic properties.

## 2. Results

### 2.1. Assessment of the Surface-Nanostructure of the Obtained Biomaterials by SEM

**Figure 1:**
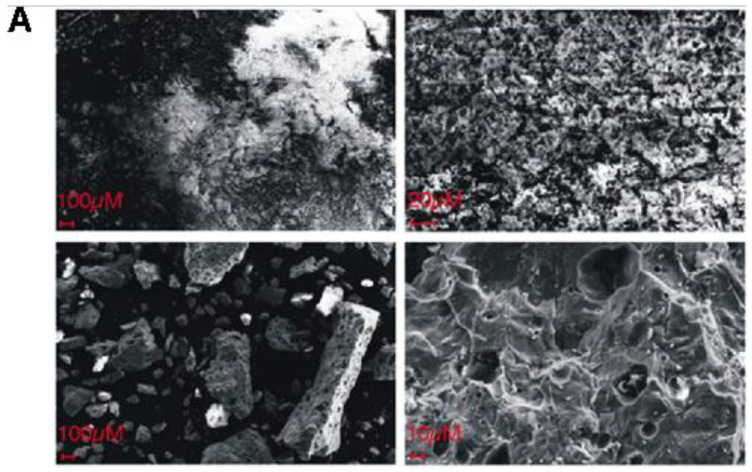

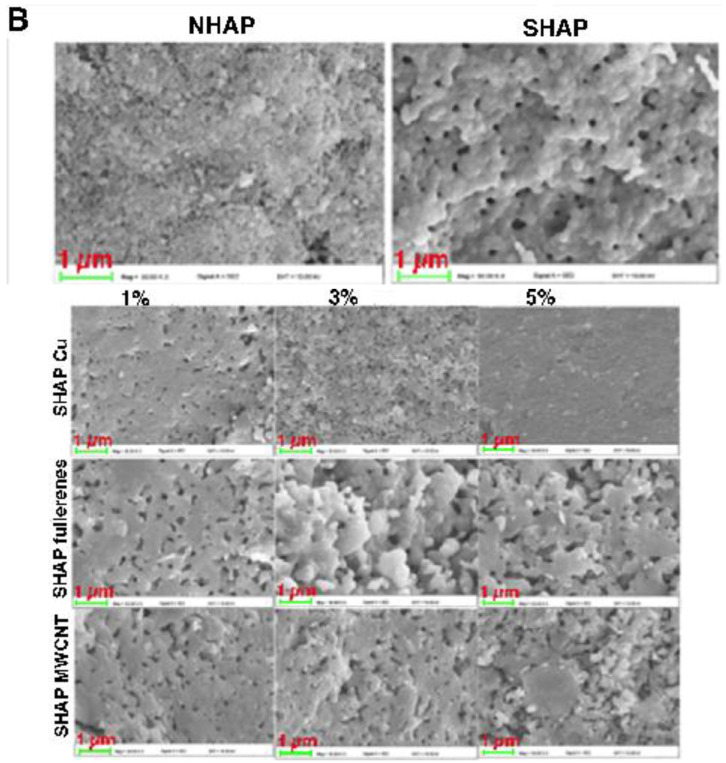
**Assessment of the surface-nanostructure of the obtained biomaterials by SEM.** (**A**) I and II (upper panels) show representative examples of ultrastructural features of SHAP. III-IV (lower panels) show representative examples of ultrastructural features of NHAP. Micrographs I and III show tested biomaterials under 300× magnification, whereas micrographs II and IV show tested biomaterials under 3000× magnification. (**B**) Upper panel shows NHAP and SHAP under 50,000× magnification, whereas the composite lower panel shows 50,000× micrographs of SHAP doped with the indicated (left side) additives, while the concentrations of the additives are shown at the top of the panel. Cu: copper nanowires.

As shown on the attached micrographs (Figure 1), the NHAP-based biomaterials showed better-developed structural features as compared to SHAP, which were largely amorphous.

Dopants used in our experiments: copper nanowires, fullerenes, and MWCNTs, are well-studied substances. Assessment of microhardness and of the electric properties of the produced biomaterials by were done by BSD.

**Figure 2:**
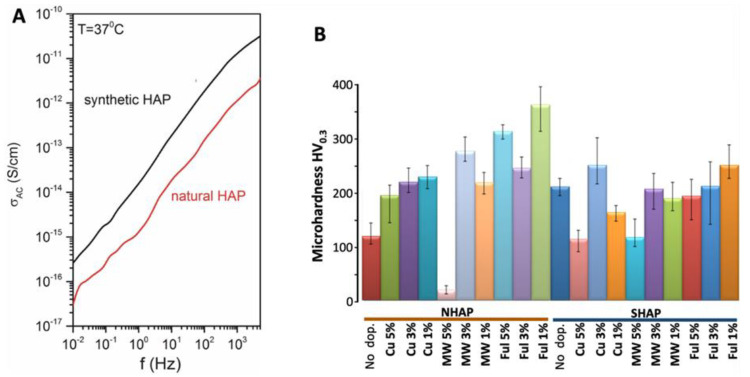
**Assessment of microhardness and of dielectric properties of the obtained biomaterials.** (**A**) The dielectric properties of NHSP and NHAP were assessed by Dielectric Spectroscopy (BSD). The measurement of AC conductivity of samples prepared from natural and synthetic hydroxyapatite was performed at a constant temperature of 37 °C. Randomly chosen example of the data is presented. (**B**) The assessment of microhardness was performed by the Vickers method. During the test, a load of 300 gf was used. No dop.: no dopants; Cu: indicates the percentage content of copper nanowires; MW: multiwalled carbon nanotubes at various concentrations; Ful.: fullerenes at the indicated concentrations. For all doped NHAP derivates, the differences in microhardness were highly statistically significant (*p* < 0.0001) when compared to non-doped NHAP. For SHAP supplemented with dopants, differences in microhardness were less pronounced, and in some instances, they were statistically non-significant. A statistically significant difference was found between non-doped SHAP and SHAP doped with copper nanowires (Cu) (*p* < 0.001). However, no statistically significant difference was found between SHAP without dopants and SHAP variants supplemented with MWCNT (MW) at concentrations of 1% and 3%, and between SHAP variants supplemented with fullerene (Ful) at concentrations of 3% and 5%. The microhardness of SHAP supplemented with 5% MW, 1% Ful, as well as with all concentrations of copper nanowires (Cu) were statistically different. A comparative analysis was performed for each material separately between different concentrations of dopant materials, and no statistically significant differences in microhardness were observed between the different concentrations except for NHAP doped with Cu 3% vs. NHAP doped with Cu 1% and SHAP between the same concentrations of MW dopants and in the case of Ful between concentrations of 5% vs. 3%. When microhardness of NHAP and SHAP was compared (between non-doped biomaterials and between biomaterials supplemented with similar dopants and at similar concentrations), the differences in microhardness were statistically significant (*p* < 0.001).

To further characterize the general properties of the obtained composites, we have next tested the (dia)electric properties of the produced biomaterials by Broad-bandpass Dielectric Spectroscopy while applying alternate currents at a broad range of frequencies. Hydroxyapatite is dielectric, which is important in implantology. During physiotherapeutic procedures, the implants would not heat up. The presented data indicate that both studied samples are insulators (Figure 2A). At the lowest measured frequency, i.e., 10^−2^ Hz, electrical conductivity is equal to 2 × 10^−17^ S/cm for natural- (NHAP) and 2 × 10^−16^ for synthetic hydroxyapatite (SHAP). Hence, contrary to the expectations, the SHAP samples were more electroconductive than NHAP ones as the natural hydroxyapatite should have more conductive additives such as ions. The higher conductivity of the synthetic sample could be related to the more compact and uniform structure formed during sintering.

We have also assessed the microhardness using the Vickers method. We measured the microhardness not only of the NHAP and SHAP but also of their doped derivates. As shown in Figure 2B, the dopants have strongly and to varying degrees affected the microhardness of the tested biomaterials. Generally, the microhardness of NHAP was strengthened by all dopants. The highest microhardness was obtained for the NHAP containing 1% of fullerenes. Only the addition of 5% MWCNT weakened the microhardness of the composite. Different results were obtained for SHAP composites. Only Cu-nanowire at a dose of 3% and fullerenes at a dose of 1% had strengthened SHAP composites’ microhardness. Other dopants slightly reduced the microhardness of SHAP biomaterials. In both cases, the addition of carbon nanotubes in the amount of 5% was unfavorable.

### 2.2. Characterization of the Thermal Properties and Stability of NHAP by DSC

**Figure 3:**
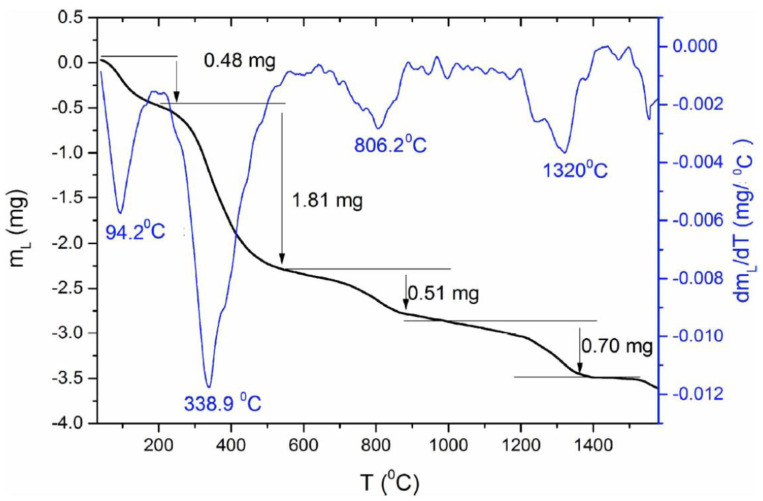
**Assessment of thermal properties of the obtained biomaterials using thermogravimetric analysis.** The thermogravimetric measurement of natural hydroxyapatite (NHAP). The black line represents the mass loss of the powder, while the blue line is the first derivative of mass loss. From the derivative peaks, one can obtain temperatures of most intensive mass losses. The data represents single sample tests. Randomly-chosen example of the data is presented.

Malina, Sofronia, and their teams have previously studied SHAP’s thermal properties [55,56]. Here, we have tested the thermal properties and stability of the produced NHAP-based biomaterial by Differential Scanning Calorimetry. As shown in Figure 3, the NHAP-based biomaterial showed a significant drop of mass in the course of heating under an argon atmosphere. Initially, the mass of the tested sample equaled 13.438 mg; then, during heating, a significant decrease in the mass of the tested sample (powdered tooth) was observed at the following temperatures: (a) 94.2 °C, samples lost 0.48 mg of their mass (3% of the initial mass); (b) 338.9 °C, samples lost 1.81 mg of their mass (13.4% of the initial mass); (c) 806.2 °C, samples lost 0.51 mg of their mass (3.7% of the initial mass); (d) 1320 °C, samples lost 0.70 mg of their mass (5.2% of the initial mass). The observed drastic mass changes were likely due to NHAP’s natural origin and associated with its traces of residual organic substances (please see the Section 3 for further details).

### 2.3. Monitoring of the Release of Selected Organic Compounds from Tested Composites to the Solvent (CDCl3) of the Obtained Biomaterials by ^1^H NMR Spectroscopy

**Figure 4:**
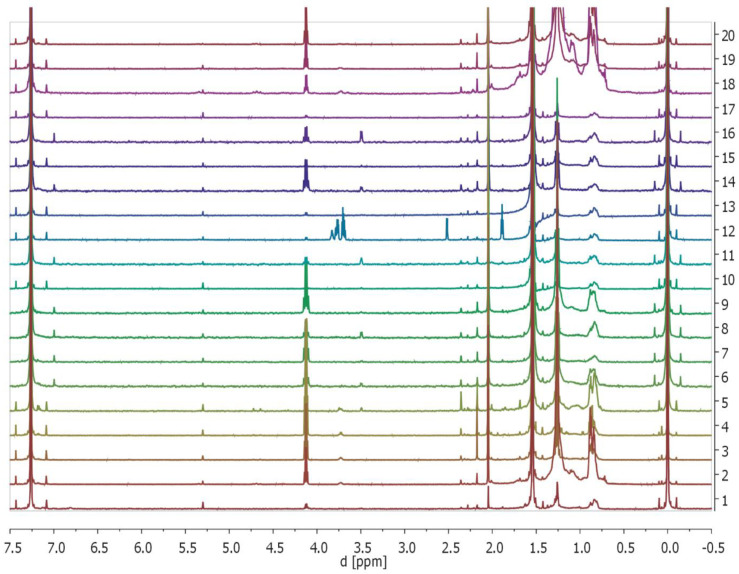
Monitoring of the potential release of selected organic compounds from tested composites to the solvent (CDCl3) of the obtained biomaterials by ^1^H NMR. The displayed NMR-spectra (numbered from bottom to top) are as follows: 1: NHAP; 2: NHAP/FUL/1; 3: NHAP/FUL/3; 4: NHAP/FUL/5; 5: NHAP/CU/1; 6: NHAP/CU/3; 7: NHAP/CU/5; 8: NHAP/MWCNT/1; 9: NHAP/MWCNT/3; 10: NHAP/MWCNT/5; 11: SHAP; 12: SHAP/FUL/1; 13: SHAP/FUL/3; 14: SHAP/FUL/5; 15: SHAP/CU/1; 16: SHAP/CU/3; 17: SHAP/CU/5; 18: SHAP/MWCNT/1; 19: SHAPMW/CNT/3; 20: SHAP/MWCNT/5 (please see the main text for peak descriptions). Randomly-chosen example of the data is presented.

To further analyze the properties and composition of the obtained biomaterials, we checked if they leach selected compounds by analyzing of extraction solvent (CDCl3) ^1^H NMR-spectra (Figure 4, please consult the Section 4 for details). In all of the recorded ^1^H NMR spectra, the following signals could be detected: internal standard—TMS (0 ppm (s, 6H)), H_2_O (1.54 ppm (s, 2H)), ethyl acetate (4.12 ppm (q, J = 7.1 Hz, 2H), 2.05 ppm (s, 3H), 1.26 (t, J = 7.1 Hz, 3H)), solvent—CDCl_3_ residual signal (7.26 ppm (s, 1H)). These are the most intensive peaks on the spectra. In addition, small signals in the range of 5.3–1.0 ppm were observed. Within this range, observed signals corresponded to aliphatic protons, alkenyl protons, or protons that were in the vicinity of the heteroatom (e.g., oxygen). This means that the samples, except for water, solvent (CDCl_3_), aliphatic compounds, and ethyl acetate, contain traces of aliphatic compounds such as ethers and alcohols. However, due to the lack of a blank sample, it is not possible to fully exclude that some of the signals come from the solvent impurities. While fullerenes and MWCNT are not protonated, the composites doped with them have also been analyzed for systematic purposes.

### 2.4. Assessment of Antibacterial Properties of the Obtained Biomaterials

**Figure 5:**
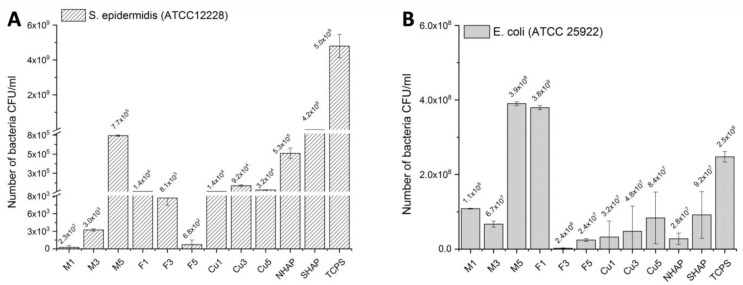
**Assessment of antibacterial properties of the obtained biomaterials.** We have assessed the adherence and survival of our composites on our biomaterials and the potential effects of dopants on two reference bacterial strains: (**A**) *S. epidermidis* (Gram-positive) and (**B**) *E. coli* (Gram-negative). M1, M3, and M5 represent, respectively, SHAP-doped with −1%, −3%, and 5% MWCNT. F1, F3, and F5 represent SHAP composites doped respectively with −1%, −3%, and −5% of Fullerenes C_60_. Cu1, Cu3, Cu5, represent SHAP doped with −1%, −3%, and −5% of copper nanowires, respectively. TCPS represents a positive control.

The antibacterial properties of the tested biomaterials were assessed using two reference bacterial strains: Gram-positive *S. Epidermidis* (ATCC 12228) and Gram-negative *E. coli* (ATCC 25922). The biomaterials were cultured with the standard concentration of the bacteria ~5 × 10^8^ CFU/mL (0.5 McFarland). After 4 h, a much lower amount of *S. epidermidis* was adhered to the investigated samples compared with the *E. coli*. As shown in Figure 5, the chemical composition of the samples significantly influenced the bacteria adhesion between the bacteria strains, as well as between the sample types. The M1 and M3 samples showed the best resistance to *S. epidermidis* adhesion. For the M5 samples, a higher amount of adhered *S. epidermidis* was recorded; however, the number of the bacteria was still relatively low. A similar amount of the adhered bacteria was recorded for the samples: Cu1, Cu3, Cu5, F1, and F3. In contrast, the F5 sample exhibited similar bacteriostatic properties as the M1 and M3 samples. No significant differences were observed between the samples incubated with *E. coli* bacteria. The numbers of recorded bacteria were similar to the reference samples. It means that the surfaces were not bacteriostatic against Gram-negative *E. coli* bacteria.

### 2.5. Biocompatibility Assessment by MTT-Assay

**Figure 6:**
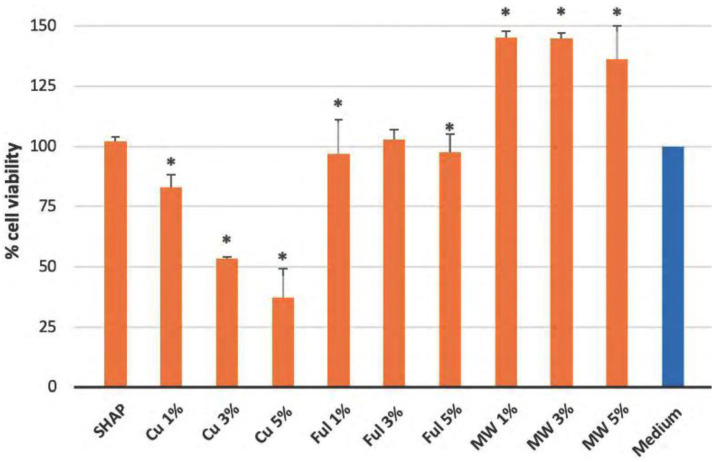
**Assessment of the biocompatibility of SHAP-based biomaterials and their doped derivates**. MW: doped with multiwalled carbon nanotubes; Ful: doped with fullerenes; Cu: doped with copper nanowires. The bars represent the percentage of cell viability as a percentage of control, that is, cells treated with PBS: medium only, where PBS was not with prior contact with any biomaterials. “*” indicates statistically-significant difference (*p* < 0.05) as compared to control (cells in 50% medium; 50% PBS).

In order to assess the biocompatibility of the tested biomaterials, we applied MTT-assay (Figure 6). The following formulas were used to calculate (i) toxicity (calculated as (sample optical density (OD_P_)/control sample optical density (OD_c_)) × 100%); (ii) percentage of growth inhibition.

(1) $$\mathrm{IP}(\%)=100(\%)-(\frac{A_{p}-A_{\mathrm{cm}}}{A_{\mathrm{cc}}-A_{\mathrm{cm}}})\times 100$$

where A_p_ = sample absorbance, A_cm_ = background absorbance of well-filled just with the cell culture medium, and A_cc_ = absorbance of wells containing untreated cells in medium, and (iii) cell proliferation as a percentage of control. Since NHAP is based on a material of limited natural origin (medical waste), we ran out of it after a single experiment. These results are shown as a Supplementary Figure S1. The presented in Figure 6 data was obtained for SHAP-based materials (no supply constraints). As shown below in Figure 6, the majority of the tested biomaterials were nontoxic. Non-doped biomaterials made of NHAP, and to a lesser degree, those made of SHAP, showed growth-promoting properties. However, marked toxicity could be observed by biomaterials doped with copper (Cu) nanowires. Comparably lower as Cu-nanowires toxicity was also observed in NHAP doped with fullerene type 1, and to a lesser degree, by NHAP doped with fullerene type 2 (Figure S1). There was no observed marked toxicity when SHAP doped with fullerenes was tested. However, marked toxicity could be observed by biomaterials doped with copper (Cu) nanowires. Comparably lower Cu-nanowires toxicity was also observed in NHAP doped with fullerene type 1, and to a lesser degree, by NHAP doped with fullerene type 2. In contrast, SHAP doped with fullerenes was in general, nontoxic.

As shown in Figure 6, the non-doped biomaterials showed no toxic properties. Non-doped biomaterials made from NHAP (Figure S1) and, to a lesser extent, non-doped biomaterials made from SHAP even showed growth-promoting properties. In contrast, pronounced toxicity was observed for biomaterials doped with copper (Cu) nanofibers at all concentrations analyzed. Lesser toxicity than for Cu nanowires was also observed for NHAP with 1% fullerenes added and to a lesser extent for NHAP with 5% fullerenes Figure S1. No apparent toxicity was observed for the fullerene-doped SHAP assay. The greatest decrease in viability was observed in the extract from SHAP with a 1% fullerene addition. In contrast, SHAP doped with 2% and 3% fullerene was generally nontoxic. Analysis of SHAP and NHAP with 1% addition of carbon nanotubes showed no toxic effect on cells. Doping of both biomaterials with higher concentrations of carbon nanotubes only marginally affected cell viability.

## 3. Discussion

**Figure 7:**
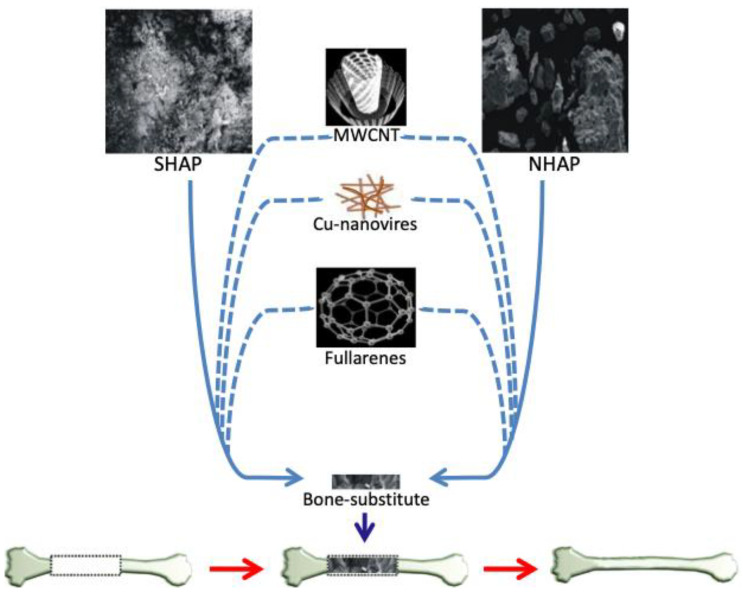
Schematic representation of materials used for the preparation of SHAP-, NHAP-based biomaterials, with the indication of the dopants.

We have prepared by sintering and initially characterized several variants of hydroxyapatite-based biomaterials, referred to below as NHAP, SHAP, and their doped derivates, as outlined in Figure 7. The sourcing of the hydroxyapatite used in the sintering process has a significant influence on the structure of NHAP and SHAP sinters observed under a scanning electron microscope (Figure 1). Previous reports indicate that the conditions under which sintering of hydroxyapatite is conducted have a profound effect on its properties. Hence, the comparison of sintering in an atmosphere of oxygen (O_2_) or in a carbon dioxide (CO_2_) atmosphere, it was proven that sintering in the presence of oxygen contributes to an increase in density and decrease of porosity as well as the chemical stability of sinters, understood as favorable conditions for the release of sodium, calcium and carbon groups as also described by Reczyński’s team [45]. On the other hand, as also previously noted by Zhang et al., sintering in a CO_2_ shielding atmosphere, which the authors used in the assay, enables maintaining higher material porosity [57]. Based on the information from the present study, a temperature of 1000 °C was applied; however, argon was used as the shielding gas instead of CO_2_. The results proved that despite applying higher temperatures, it was possible to obtain porous sinters (Figure 1).

Next, we have studied both the antimicrobial properties of the obtained composite materials (on standard strains) as well as the physical properties. We have assessed samples’ microhardness to determine the influence of the additive (organic or inorganic) on the obtained microstructure under different percentage shares (1–5%). Significant differences in microhardness, even for non-doped NHAP and SHAP, were observed. The highest microhardness was achieved for composite materials based on NHAP. The SHAP sinters were created out of globular nanoparticles of synthetic hydroxyapatite with a diameter of 20–30 nm. On the other hand, NHAP sinters were created out of teeth. As communicated, i.e., by Zheng et al. [58], NHAP is composed of two principal parts: (i) crown, which is characterized by a higher amount of inorganic material, which means high hardness, and (ii) root, with higher content of organic material (~20%), about 10% of water, and is responsible for mounting the whole tooth in the periosteum. Interestingly, the introduction of dopants like carbon nanotubes (MWCNT), fullerenes (FUL), and copper nanowires (Cu), noticeably increased microhardness when dopants were present in the quantities of 1% and 3% for NHAP-based composites. In comparison with synthetic hydroxyapatite, for doped SHAP, no satisfactory results were obtained. Both NHAP and SHAP doped with 5% Cu or MWCNT experienced a significant hardness-decrease, which may be due to lower residual hardness of the used dopants (as compared to hydroxyapatite), that at 5% became evident for the tested doped sinters.

Stiffness of the tissue microenvironment favors stem cell differentiation in certain directions, including osteoblastoid. The used dopants allow for fine-tuning of the composite hardness, which may need to be adjusted when seeking optimal bone replacement materials.

MWCNTs are frequently used as dopants due to their interesting biophysical properties. Here we have doped SHAP or NHAP matrix with MWCNT in order to determine their impact on the structure and biological properties of the composite biomaterials materials obtained.

In a similar context, we doped our experimental hydroxyapatite-based biomaterials with copper nanowires. Copper is a chemical element from the group of transition metals that is widespread in nature. In its pure form, copper oxidizes quickly, and in excessive amounts, it can be toxicl therefore, the current work is focused on the use of copper compounds in order to reduce its toxicity, e.g., by combining it with calcium phosphates but also with silicate glasses [59,60,61]. Research was also carried out on the use inclusion of copper in the development of bioactive tissue scaffolds. Copper nanoparticles or nanowires are characterized by a large surface to mass ratio; therefore, after combining with polymer solutions, they can be transformed into micro and nanofibers, i.e., when combined with, among others, nanocellulose and lignin. In this form, nano-copper is tested for the prevention of dermatitis [62]. The production of biomaterials containing a combination of silver and copper has been reported, which promotes the synthesis of hydroxyapatite, the mineral building block of bone tissue [63]. Copper combined with pectins and transformed into nanofibers is tested for carriers of medicinally-active substances [64]. The antibacterial properties of copper, its protective effects on the cardiovascular system, supportive role in the healing of bone fractures and cut wounds increase the interest in this chemical element. Copper plays a significant role in positron emission tomography (PET) imaging, radioimmunoassay, and cancer radiotherapy. Due to the numerous biological functions of copper, researchers are currently focusing on the development of new biomaterials containing this metal [65,66]. Current research focuses on the role of copper in cancer. Tumor growth and metastasis are associated with an increased demand for this metal. The identification and characterization of new copper-dependent signaling pathways, hyper-activated in cancer, offers great therapeutic potential [67].

As already mentioned in the introduction, copper has been tested for new antimicrobial packaging materials [52] and has been under consideration in glucose sensors [53], but also as a transparent conductor with increased thermal stability [54].

As summarized by Bosi’s and De Ros’s teams, fullerenes are the third allotropic form of carbon. Because they are entirely made of carbon, similarly to carbon nanotubes, they exhibit hydrophobic/lipophilic properties, thus enabling integration into the biological membranes, and they may lead to their destabilization [68], which translates into antimicrobial properties of fullerenes and salts generated with them [69]. Both carbon nanofibers and fullerenes, although entirely made of carbon, differ in the spatial arrangement of carbon atoms; cylindrical for nanotubes and spherical for fullerenes. These spatial arrangements of carbon molecules affect their properties. Hence, we have used both allotropic forms of carbon to compare their properties when incorporated into the hydroxyapatite matrix. As evident from the testing in Figure 5, fullerenes at a concentration of 1%, and to a lesser degree, fullerenes at a concentration of 3%, both exhibited bacteriostatic properties against the reference *S. epidermidis* strain. The MWCNT samples doped with 1% and 3% also were bacteriostatic against Gram-positive bacteria. The interpretation of those observations is difficult, especially as the sintering by itself (exposure to high temperature) may have caused structural changes to both allotropic forms of carbon. The final effects of the bacteriostatic properties depend, for example, on the surface morphology, chemical composition, surface roughness, and wettability. In our case, the chemical composition and the amount of copper ions present at the top of the samples played a key role. Initial adhesion of the bacteria on the sample surface is observed after several seconds of bacteria culture. Four hours of the bacteria culture allowed us to observe the bacteria adhesion and protein productions, which caused the formation of the biofilm. The activity of the Gram-positive and Gram-negative bacteria is also different. *E. coli* bacteria is larger than *S. epidermidis* and grew faster in the culture medium. In our case, the material modifications of the MWCNT and the fullerenes (F5) improved their bacteriostatic properties against *S. epidermidis*. The surfaces of the samples did not significantly inhibit *E. coli* adhesion but also were not favorable for their growth, which was observed in the reference sample (TCPS).

In terms of electric properties, the results of tests with the use of BSD clearly indicate that although both NHAP- and SHAP-based matrixes are dielectric, they exhibit differences in electric conductivity. The chemical purity of both of the materials has a significant influence on the difference in the obtained conductivity. Synthetic hydroxyapatite (SHAP) is a nanopowder with a diameter of 20–30 nm, whose purity equals 99.99%, while natural hydroxyapatite NHAP contains tissue-forming elements which make up the tooth, including enamel as well as pulp. As previously noted by Zheng et al. [58], these content differences likely explain the observed difference in conductivity of both samples. A high temperature of 1000 °C was used in the study, along with a shielding atmosphere of argon. Such high temperature removes organic impurities. The tissue elements were damaged, which could have also increased the purity of the material and, at the same time, reduced the conductivity of the tested NHAP samples (Figure 2).

During the heating of the NHAP sample in the argon atmosphere, a significant drop in the mass of the tested sample was observed. Initially, the mass of the tested sample equaled 13.438 mg. As a result, after heating, the sample weighed 9.938 mg, which means a decrease in the initial mass of the sample by 26.1%, and that is the value that should be assumed as far as a decrease in the mass of the samples sintered to the temperature of 1500 °C. The observed decrease of mass may be connected with loss of water, especially at the temperature of 94.2 °C, as well as a breakup of organic and inorganic particles at temperatures of 338.9 °C (organic-associated water), 806.2 °C (loss of structure-trapped water), and 1320 °C (loss of CO_2_ due to decarboxylation), which are a result of the breakup of, among others, enamel and pulp (Figure 3). The obtained thermogravimetric data follows, in general, the pattern previously published by Gómez-Gasga’s team [70].

Tests conducted with the use of NMR spectroscopy have shown the presence of trace amounts of compounds of organic origin. Their presence was observed in all the tested composite materials, both those created on the basis of synthetic hydroxyapatite SHAP as well as those on the basis of natural hydroxyapatite NHAP. However, a significantly higher amount of organic substances could be observed in composites created on the basis of NHAP, which is likely due to the organic structures that make up the tooth.

Among tested dopants, we also included copper nanowires. In the context of nanomaterials, copper nanowires are most often tested with regard to transparent conductive materials. As highlighted by the team of Sun, and Zheng, copper, due to its properties, is also tested when new alloys with antimicrobial properties are sough [54,58]. Our experiments revealed only moderate antibacterial properties of hydroxyapatite doped with copper nanowires, which may reflect a relatively low concentration of Cu-ions in the composite. All tested biomaterials, also the doped variants, showed no, or only marginal toxicity towards the normal human fibroblasts used as indicator cells. The highest toxicity was observed by biomaterials doped with copper nanowires in a dose-dependent manner. Both non-doped biomaterials exhibited even growth-promoting properties, likely due to the extra-availability of Ca^2+^ ions.

## 4. Materials and Methods

### 4.1. Materials

The following materials were used: synthetic hydroxyapatite with a particle diameter of 20–30 mm from SSnano (Houston, TX, USA), in the text, referred to as SHAP, natural hydroxyapatite from biological material (discarded teeth, and teeth fragments in bulk, considered a medical waste), supplied from the Medical University of Silesia in Katowice, which are in the text referred to as NHAP. In Poland, the use of discarded human material (medical waste) does not require ethical approval, only notification of the local Ethical Board, which was dully done. Multiwalled carbon nanotubes (MWCNT) with a diameter of 50–100 mm, fullerenes C60-C70, and Cu nanowires with a diameter of 300 mm were all from SSnano. CDCl_3_ for NMR was Trypsin-EDTA.

### 4.2. Sintering of Composite Materials with SHAP Matrix

In order to obtain composite materials with a synthetic hydroxyapatite (SHAP) matrix, test portions of hydroxyapatite were prepared, then the following were added to each individual test portion: (i) carbon nanotubes in the amount of 1% (SHAP/MWCNT/1), 3% (SHAP/MWCNT/3), and 5% (SHAP/MWCNT/5), or (ii) Cu nanowires in the amount of 1% (SHAP/CU/1), 3% (SHAP/CU/3), and 5% (SHAP/CU/5), or (iii) fullerenes in the amount of 1% (SHAP/FUL/1), 3% (SHAP/FUL/3), and 5% (SHAP/FUL/5). Then the materials were mixed with the use of a ball mill; 10 grinding cycles were used for each portion, one cycle consisted of 1 min of grinding and a 2 min break, and the speed of mechanical grinding was set at a level of 450 rotations per minute. After grinding, the samples were dried at a temperature of 30 °C for 24 h. After drying, test samples were prepared, which were pressed in the form of pills with a diameter of 1 cm. They then underwent sintering in a shielding atmosphere of argon. The heating process was set at 5 °C/min; the sintering process lasted 1 h at a temperature of 1000 °C, then the cooling process took place until the temperature of 25 °C.

### 4.3. Generation of Composite Materials with NHAP Matrix

Teeth are composed not only of enamel (mostly hydroxyapatite with traces of water and organic material) but predominantly of dentin (hydroxyapatite with substantial collagen, organic material, and water content) and cement of a comparable composition like dentin. For our study, the NHAP was sourced from discarded teeth (medical waste) provided by the Department of Craniomaxillofacial Surgery, Faculty of Medical Sciences in Zabrze, Katowice, Poland, headed by Prof. Iwona Niedzielska. Biologic hydroxyapatite (NHAP) is less pure; it contains carbonate, and other ions, which substantially change its properties. In order to obtain composite materials with natural hydroxyapatite (NHAP) matrix, firstly, the biological material was degreased and dehydrated with the use of ethanol solutions, 50–70%. Then it underwent initial fragmentation, and the materials prepared in such a way were dried at room temperature for a time of 24 h. Subsequently, the biological material was ground. After grinding, it was once again dried at room temperature for 24 h. From the dried powder, hydroxyapatite test portions were prepared to which the following were added: (i) carbon nanotubes in the amount of 1% (NHAP/MWCNT/1), 3% (NHAP/MWCNT/3), and 5% (NHAP/MWCNT/5), or (ii) Cu nanowires in the amount of 1% (NHAP/CU/1), 3% (NHAP/CU/3/), and 5% (NHAP/CU/5), or (iii) fullerenes in the amount of 1% (NHAP/FUL/1), 3% (NHAP/FUL/3), and 5% (NHAP/FUL/5). Then the materials were mixed with the use of a ball mill; 10 grinding cycles were used for each portion, one cycle consisted of 1 min of grinding and a 2 min break, and the speed of mechanical grinding was set at a level of 450 rotations per minute. After grinding, the samples were dried at a temperature of 30 °C for 24 h. Then, test samples were prepared, which were pressed in the form of pills with a diameter of 1 cm. The sintering was conducted under an argon shielding atmosphere. The heating process was set at 5 °C/min; the sintering process lasted 1 h at a temperature of 1000 °C, then the cooling process took place until the temperature of 25 °C.

### 4.4. Scanning Electron Microscopy

The structure of composite materials was assessed by SEM, by a ZEEIS SUPRA 25 SEM scanning microscope, using accelerated voltage 3–25 kV. The observations were made with magnifications of 1000–100,000 times in order to determine the sample surface.

### 4.5. Differential Scanning Calorimetry

The mass loss in hydroxyapatite during heating was measured by DSC by means of a Netzsch STA Jupiter F3 analyzer, which enables simultaneous analyzing the mass of the sample and calorimetric properties. The measurement was performed with the heating rate of 2 °C/min, with argon atmosphere (argon flow 100 mL/min) in Al_2_O_3_ crucibles. The sample was heated in the rhodium furnace from RT to 1600 °C.

### 4.6. Nuclear Magnetic Resonance

NMR was used to monitor the release of selected organic compounds from tested composites to the solvent (CDCl3). NMR spectra were recorded with a Varian spectrometer 600 MHz using TMS as an internal standard and CDCl_3_ as a solvent. NMR solvent was purchased from ACROS Organics (Geel, Belgium). Chemical shifts (δ) were expressed in ppm and coupling constants (*J*) in Hz.

### 4.7. Microhardness

Microhardness measurements were conducted using the Vickers method, using the FUTURE-TECH FM-ARS 9000. Prior to the measurement, the surfaces of the samples were abraded (without the usage of water) with a paper with a gradation of 4000 (approximately 5 µm) as well as cleaned with compressed air. During the test load of 300 gf was used.

### 4.8. Antimicrobial Tests

The bacterial strains used throughout the experiments were *Staphylococcus epidermidis* (ATCC 12228) and *Escherichia coli* (ATCC 25922). These bacteria were precultured in a TSB (tryptic soy broth (Tryptone (Pancreatic Digest of Casein)) 17.0 g/L, Soytone (Peptic Digest of Soybean) 3.0 g/L, Glucose 2.5 g/L, Sodium Chloride 5.0 g/L, Dipotassium Phosphate 2.5 g/L), Biomaxim, Poland) culture medium (Graso, Biotech, Poland) at 37 °C for 18 h (incubator CLW, POL-EKO). The samples were in 24-well plates (VWR), sterilized under UV lamp for up to 30 min, and then 1 mL of bacteria (~5 × 10^8^ CFU/mL) was seeded. The bacteria were cultured for 4 h at 37 °C. After 4 h, an inoculum with bacteria was removed, and the samples were carefully washed using a sterile water solution. Then, the samples were transferred to new 24-well plates and were shaken several times using 1 mL of 0.25% trypsin in a phosphate-buffered saline w/o Ca and Mg (Biowest U.S.). 100 μL of the solution was added to the diffusion agar plates Diag-Med, Poland) in a concentration of solution that was mixed with sterile 0.9% NaCl (1:0, 1:1, 1:10, 1:100, 1:1000, 1: 10,000, 1:100,000). The agar plates were incubated for 18 h at 37 °C, and then the bacteria strains were counted.

### 4.9. Biocompatibility Assessment

The effects of obtained biomaterials on cell survival and proliferation were assessed by 3-(4,5-dimethyl-2-thiazolyl) 2,5-diphenyl-2H tetrazolium bromide (MTT) assay. The biomaterials were soaked in PBS for 24 h. Such PBS solutions were used for MTT assay upon dilution 1:1 in fresh complete DMEM-F12 medium containing 10% FBS (hereinafter referred to as “1:1-diluted PBS”). The 1:1-diluted PBS that was previously in contact with the tested biomaterials was then used for toxicity assessment using normal human fibroblasts (NHDF) as test cells. NHDF (Gibco™ Cat. #: C0135C) were seeded at 1 × 10^4^ cells/well, in a flat-bottom 96-wells plate in fresh DMEM-F12 medium, supplemented with 10% FBS, 24 h prior to the assay. The 1:1-diluted PBS samples we then incubated for the next 72 h without further dilutions with NHDF to assess any possible toxicity (inhibition of proliferation). Next, the 1:1-diluted PBS was removed, and cells were washed with PBS solution. Then, each well was filled with 20 µL (5 mg/mL]) MTT solution and incubated in the cell culture incubator for 3 h. Next, the plates were centrifuged, and the supernatant was discarded. Formazon crystals were dissolved in isopropanol-HCl solution (1:1 ratio). The readings were performed at 570 and 630 nm using a spectrophotometer (Epoch, TKBiotek, Biokom, Janki, Poland).

### 4.10. Statistical Analysis

The statistics were calculated on the values obtained by initial statistical analyses, i.e., average, SD, minimum, and maximum. Homogeneity of variances estimated using Levene’s test or Bartlett’s test. For comparison between groups, a Student’s *t*-test or its non-parametric equivalent, the Mann–Whitney U test, was used. For comparison to the control group used, a Dunnett test or its non-parametric equivalent, a Steele’s test, was used. Comparative analysis for each of the analyzed biomaterials between the different concentrations was used using an ANOVA test or Kruskal–Wallis test. As a post-hoc test, Tukey’s HSD or Steel-Dwass tests were used. The statistical difference between groups has been assessed at the level of *p* ≤ 0.05. Analysis was carried out using programs Statistica 13.3 and KyPlot (Kyence Inc., Tokyo, Japan).

## 5. Conclusions

The tested dopants offer fine-tuning options, allowing for potentially adapting composites’ properties to the specific therapeutic needs. The samples showed bacteriostatic properties against *S. epidermidis* (ATCC 12228) bacteria, which confirms the hypothesis guiding the study design, whereas they did not significant inhibit the adhesion of *E. coli* (ATCC 25922). This property may offer an additional application for our composites, namely as a tool for preferential removal of certain types of bacteria from the environment under treatment.

## Supplementary Materials

The following supporting information can be downloaded at: https://www.mdpi.com/article/10.3390/ijms23094692/s1.
