# Supplementary material for: Comparison of Physicochemical, Mechanical, and (Micro-)Biological Properties of Sintered Scaffolds Based on Natural- and Synthetic Hydroxyapatite Supplemented with Selected Dopants
Source: Int J Mol Sci. 2022 Apr 23;23(9):4692. doi: 10.3390/ijms23094692 (PMC9101299; doi:10.3390/ijms23094692)
Supplement: Supplementary file 1 [file ijms-23-04692-s001.zip › ijms-1689530-supplementary.pdf]

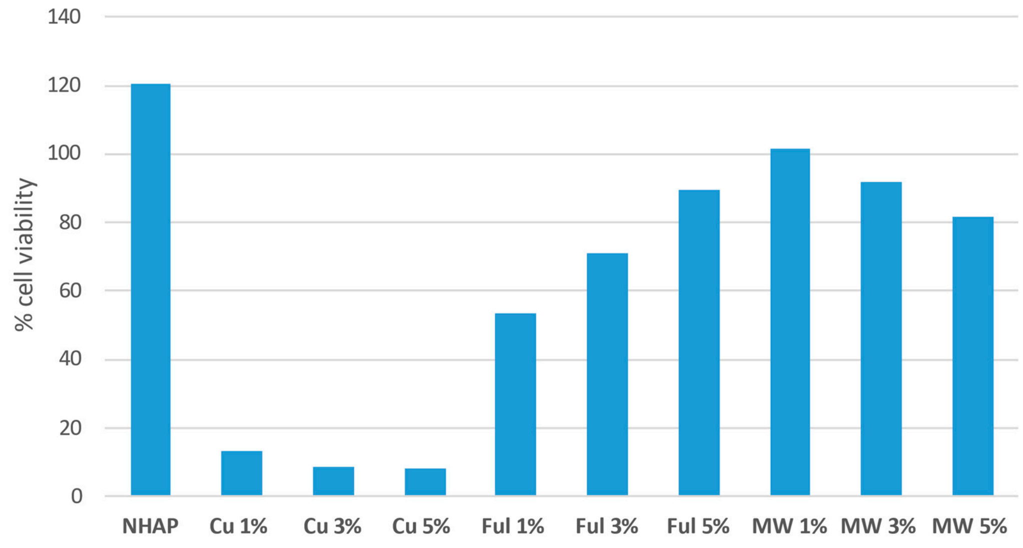

Figure S1. Assessment of the biocompatibility of NHAP-based biomaterials and their doped derivates. MW = doped with multiwalled carbon nanotubes; Ful = doped with fularenes; Cu = doped with cooper nanowires. The bars represent percentage of cell viability as percentage of control, that is cells treated with PBS:medium only, where PBS was not with prior contact with any biomaterials. Due to the lack of sufficient quantity of NHAP-hydroxyapatite of natural origin, the experiment was performed only once.
